# Quantitative evaluation of electrographic response to electroconvulsive therapy in super-refractory status epilepticus

**DOI:** 10.3389/fneur.2024.1493336

**Published:** 2024-12-16

**Authors:** Raphaël Christin, Harrison Hines, Lauren Hophing, Ankit N. Khambhati, Edilberto Amorim, Manu Hegde, Elan L. Guterman, Jonathan K. Kleen

**Affiliations:** ^1^Department of Neurology, University of California, San Francisco, San Francisco, CA, United States; ^2^Department of Neurological Surgery, University of California, San Francisco, San Francisco, CA, United States; ^3^Weill Institute for Neurosciences, University of California, San Francisco, San Francisco, CA, United States

**Keywords:** epilepsy, neurocritical, encephalitis, NORSE, quantitative

## Abstract

**Objective:**

Electroconvulsive therapy (ECT) has been occasionally applied as a treatment for super-refractory status epilepticus (SRSE). However, the effects of ECT on electrographic activity and related clinical outcomes are largely unknown. Here, we use quantitative approaches on electroencephalography (EEG) data to evaluate the neurophysiological influences of ECT and how they may relate to patient survival.

**Methods:**

This was a single center study of adult patients who underwent bi-frontal ECT for treatment of SRSE between 2007 and 2021. Continuous scalp EEG data obtained before and after each ECT session was converted using a linelength transform and projected into low-dimensional space using complementary linear and non-linear dimensionality reduction techniques (principal component analysis and separately uniform manifold approximation). Differences between before versus after ECT were quantified using silhouette scores. Mixed effects models evaluated whether changes in mean scores were related to time (across sessions, and separately within sessions up to 1 h after treatment) and patient outcomes (survival).

**Results:**

Eight patients underwent ECT for SRSE, ranging from 3 to 12 sessions each. Four patients survived with chronic epilepsy and varying cognitive sequelae, and four died while hospitalized. Projecting EEG data into low-dimensional space revealed several sessions with visualizable differences in electrographic activity before versus after ECT treatment. Silhouette scores significantly increased as time elapsed up to 60 min after ECT and higher scores were related to survival, though there was no significant change in scores across successive ECT sessions.

**Discussion:**

ECT is associated with changes in electrographic activity in certain patients, and such changes may be associated with survival, although our study was underpowered to detect more definitive treatment-related effects. Further quantitative neurophysiology studies, and potentially clinical trials, in larger groups of patients are warranted to study direct influences of ECT treatment given the devastating and often deadly outcomes of SRSE.

## Highlights

- Electroconvulsive therapy (ECT) is sometimes used as an alternative treatment for super-refractory status epilepticus (SRSE), yet quantitative evaluations of its influence are lacking.
- Dimensionality reduction and unsupervised clustering were used to compare scalp EEG signals before and after ECT stimulation in eight patients with SRSE.
- ECT during ongoing SRSE was associated with changes in EEG signal composition in a subset of sessions.
- Electrographic changes associated with ECT were marginally related to outcomes (surviving) after SRSE, though further studies with larger patient volumes are needed.

## Introduction

Super-refractory status epilepsy (SRSE) is a state of continuous or intermittent seizures that persists or recurs despite treatment with appropriate anti-seizure medications and at least 24 h of appropriate anesthetic therapy. SRSE has a roughly 24% in-hospital mortality rate (1). Nearly all survivors experience multi-organ injury and long-term morbidity as a result of relentless ictal activity and potential iatrogenic effects of prolonged use of anesthetics and anti-seizure medications. Comorbidities among survivors include permanent cognitive impairment, psychiatric disease, and other neurological symptoms, including high rates of epilepsy (2). New and better therapies are clearly needed for this devastating condition.

Electroconvulsive therapy (ECT) has been used as treatment for SRSE but evidence for its benefit is limited to case reports and small case series (3–17). Conceptually, the electrical stimulation pulses in ECT are thought to disrupt and “reset” neurophysiologic circuits to help mitigate continuous seizure activity (18). However, despite investigations into ECT effects on scalp EEG parameters in psychiatry (19), the electrographic (and by extension the related neurophysiological) effects of ECT on SRSE are poorly described. Specifically, the degree to which ECT induces electrographic changes, and whether such changes impact clinical outcomes, are unknown (18).

Quantitative studies of scalp EEG data (20–23) are increasingly used to provide an objective assessment of neurophysiological changes. While surveying general changes in EEG background patterns is valuable (16), emerging computational techniques may be better suited for the stark challenges of accounting for the complexities of varying waveform features across multiple electrodes for extended time periods (20, 23). We aimed to evaluated electrographic changes associated with ECT among patients with SRSE using such techniques, and to determine whether these electrographic changes could be used to predict clinical outcomes.

## Methods

### Study design and subjects

This was a self-controlled case series study to examine electrographic changes associated with ECT among patients with SRSE. Retrospective chart and EEG review identified all patients at a single academic center who met the inclusion criteria of (1) underwent ECT for SRSE between January 1st, 2007, and December 31st, 2021, and (2) had digital EEG data available both pre- and post-stimulation from at least one ECT session. This study was approved by the Institutional Review Board via the UCSF Committee on Human Research including a waiver of consent on the de-identified retrospective data analyzed herein.

### Clinical variables

**Figure 1:**
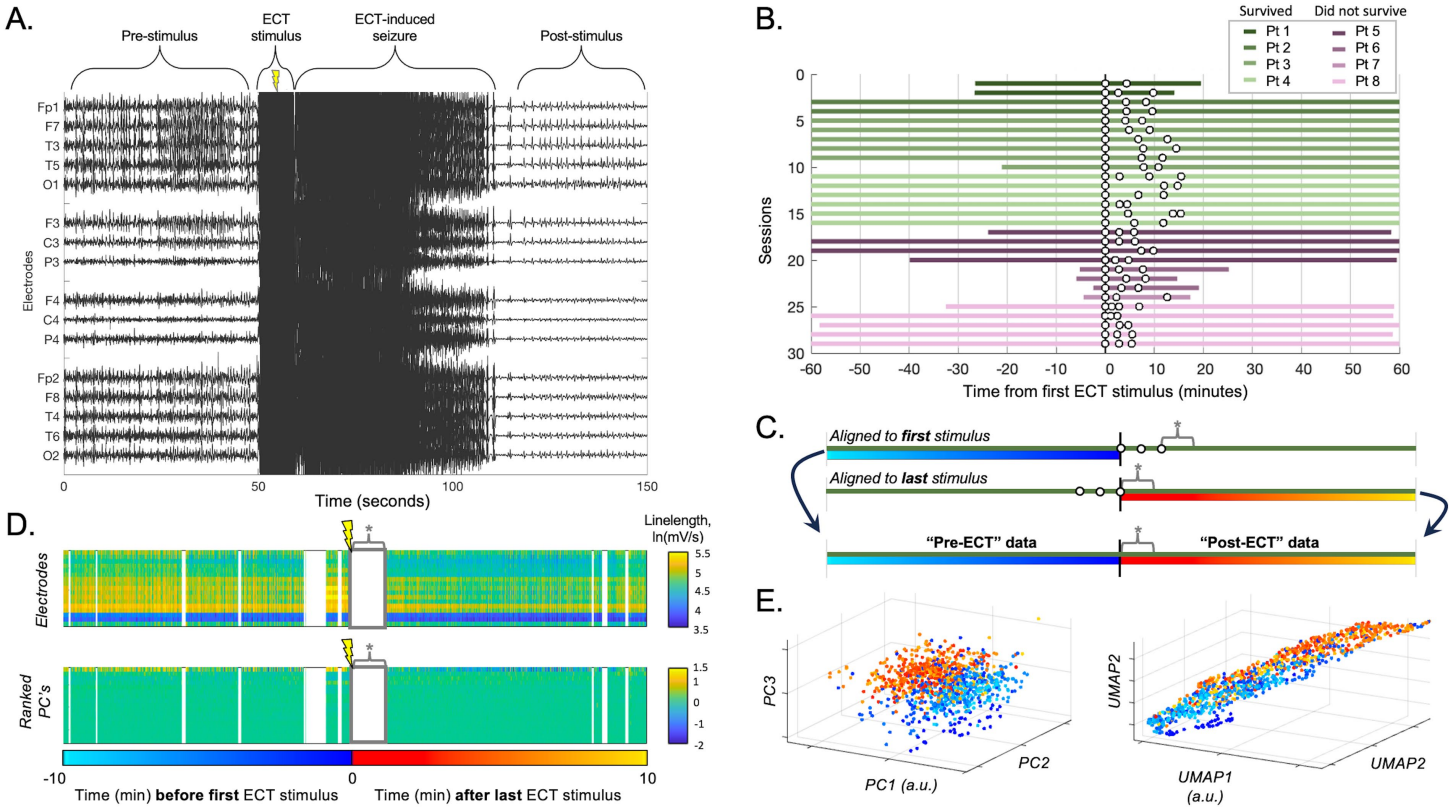
ECT stimulation session data processing. **(A)** Raw scalp EEG data from an example ECT stimulation during an ECT session, demonstrating epileptiform activity before and after the stimulation artifact (along with the temporary superimposed increase in seizure activity induced by ECT). The ECT stimulus artifact and temporary ECT-related seizure data were omitted from analyses to focus on more durable effects of ECT. **(B)** Available data for all included patients. Each row designates an ECT session with up to 60 min before and after ECT, with individual ECT stimuli as white dots (note: plot shows data aligned to first stimulus, though post-ECT data for all subsequent analyses was aligned to the last stimulus as time zero). **(C)** Schematic of session data realignment for all subsequent analyses. The “pre-ECT” data is obtained from the 60 min prior to the first stimulus, whereas the “post-ECT” data is obtained from the 60 min after the last stimulus. Data between stimuli was avoided due to variability in number and timing of stimuli across sessions. Asterisk (*) denotes ECT-induced seizure annotated for each individual session (omitted from subsequent analyses). **(D)** The upper panel shows the natural log of the linelength transform of EEG data over time (x-axis) for each channel (y-axis). Data omitted due to EEG artifact is in white vertical sections, and data omitted due the initial ECT-induced seizure is labeled * as in C. The lower panel showed the same data converted into principal components (PCs), ranked by the percent variance explained (from top, 78.1, 6.4, and 4.5% for PC 1, 2, and 3 respectively). **(E)** Data according to the top three PCs from D is plotted in 3-D space (each data point = 1 s; turquoise-blue = pre-ECT, red-orange = post-ECT), demonstrating a subtle separation in this space between pre- vs. post-ECT stimulation in that session. Right panel shows same data similarly processed with UMAP (first three dimensions).

We reviewed the electronic medical record and EEG database to collect patient age, sex, race, anesthetic and antiseizure medications used during the hospitalization, in-hospital survival, 90-day clinical outcome, 1-year clinical outcome, and date/time of each ECT stimulus. We confirmed the diagnosis of SRSE by reviewing clinical notes and the continuous EEG recordings (Figure 1A). ECT was administered over multiple sessions for each patient, and each ECT session included the delivery of two or more electrical stimuli spaced approximately 3 min apart on average (Table 1; Figure 1B; further reliable details of the specific ECT parameters were unfortunately not available in the retrospective medical record).

**Table 1:** Demographics and pertinent information for the eight patients.

ID	Outcome	Age (y)	Sex	Imaging	Lumbar puncture	Onset to adm. (d)	Onset to ECT (d)	ECT to discharge (d)	Outcome (90 d)	Outcome (1 y)	# ECT sessions	Usable sessions	Average stim./session	# anes-thetics	# of ASMs
1	Discharged	27	M	Normal	Abnormal	3	23	23	Epilepsy, cognitive symptoms	Epilepsy	5	2	3	3	7
2	Discharged	32	F	Abnormal	Normal	N/A	N/A	13	Epilepsy	Epilepsy	4	2	2.7	N/A	4
3	Discharged	23	F	Normal	1 of 2 abnormal	33	41	33	Epilepsy, cognitive symptoms	Epilepsy, cognitive symptoms	11	8	2.7	3	6
4	Discharged	30	M	Abnormal	Normal	6	10	41	Epilepsy, cognitive symptoms	Epilepsy, cognitive symptoms	12	12	3.2	3	6
5	Deceased	20	M	Abnormal	Abnormal	5	9	21	Deceased	Deceased	5	4	3	3	4
6	Deceased	19	M	Normal	Abnormal	2	18	14	Deceased	Deceased	5	5	3	4	3
7	Deceased	28	F	Abnormal	Abnormal	0	17	N/A	Deceased	Deceased	3	1	3	3	5
8	Deceased	67	F	N/A	N/A	N/A	N/A	N/A	Deceased	Deceased	3	3	3.3	N/A	N/A

The term “discharged” means that the patient was eventually transferred back to the original admitting hospital following clinical improvement. The total number of ECT sessions performed before discharge is in the “Total sessions” column, while the number of sessions with pre- and post-stimulation EEG data available (not deleted) is in the “Usable sessions” column. y, years; d, days; adm., admission; stim, ECT stimuli; ECT, electro-convulsive therapy; ASMs, anti-seizure medications used during hospitalization; N/A, not available (data not available in the electronic medical record).

### EEG recordings

EEG data were recorded using Nicolet Inc. (2007–2013) or Natus Inc. (2014–2021) data acquisition systems. All EEG recordings were performed using a standard 10–20 montage. We exported EEG data 1 h before and 1 h after each ECT session (Figures 1A,B). Due to digital storage limitations, certain portions of scalp EEG recordings (most often those obtained prior to infrastructure upgrades in 2019) were pruned (deleted) and unavailable for analysis. The number of total and available sessions is included in Table 1, and the duration of data available for individual sessions is illustrated in Figure 1B. We included up to six ECT sessions for each patient in the analysis.

Artifact is a common problem in critical care scalp EEG recordings (24), which prevents reliable quantitative analyses of the EEG data due to contamination with spurious values which can heavily skew data (25). To address EEG artifact in our dataset, each EEG recording was annotated by trained epileptologists (L.H., J.K.K.). Segments of the recordings with temporary artifacts (e.g., due to bedside care, muscle artifact) and artifact from ECT stimulation (Figure 1A) were marked and omitted from analysis (24). Channels that were not recording neural signal or were otherwise unreliable in the analysis periods (e.g., due to intermittent or persistent impendence issues) were removed from the analysis. This study targeted any potential lasting effects of ECT rather than temporary ECT-induced seizures. Therefore, any temporary overt ECT-induced seizures superimposed on the background of SE were also annotated and omitted. A temporary ECT-induced seizure was defined as a rapidly evolving superimposed ictal discharge pattern that occurred immediately after ECT stimulation and lasted less than 2 min (Figure 1A).

### Signal processing

Recordings were originally digitized as referential recordings (to CPz) at 256 or 512 Hz, and the latter were downsampled to 256 Hz preceded by an anti-aliasing filter (<127 Hz low-pass Butterworth filter). Notch filters were applied (60 Hz and harmonics) (20). Data for each channel in each session was converted using a linelength transform, calculated by summing the absolute value of the signal derivative, which has been shown to have good sensitivity and specificity as a surrogate measure for epileptiform activity (26, 27). The linelength transform was performed with a one-second window that was slid datapoint-by-datapoint to preserve time resolution. These data were converted into consecutive one-second data points by taking the mean across every 256 timepoints (non-overlapping one-second windows). We then applied a natural log transform to approach normality of the data distribution. All data processing and subsequent analysis was performed in MATLAB (MathWorks, Inc., Natick, MA, USA).

### Data visualization and statistical analysis

To address the complexity of analyzing EEG signals across up to 19 different electrodes over time, we applied two different dimensionality reduction approaches. First, we applied principal component analysis (PCA), a linear reduction technique, by transforming the data from the peri-ECT window (segments before the *first* stimulus and after the *last* stimulus of each ECT session, as outlined below and in Figures 1C,D) into its principal components. We then projected the data in 3D coordinate space of the first, second, and third principal components (Figures 1D,E) to visualize electrographic differences in the EEG recording before and after ECT as graded colors and enable cluster analysis using these same three dimensions. Second, as a non-linear reduction technique to better preserve the global structure of the data relative to PCA, we applied uniform manifold approximation and projection (UMAP) (20, 28, 29). Parameters used were a three-dimensional Euclidean embedding and neighborhood of 15, and the results were plotted similar to PCA to visualize and analyze electrographic differences in the EEG recording before and after ECT in UMAP embedding space (Figure 1E, right panel). Importantly, the PCA and UMAP embeddings were computed using unlabeled data (“blinded”) in order to reveal any latent structure to the data, which we hypothesized may arise due to the influence of ECT.

To evaluate the association between ECT and changes electrographic activity using PCA and UMAP projections in low-dimensional space, our aim was to compare electrographic activity *before* each ECT session (pre-ECT) to electrographic activity *after* each ECT session (post-ECT). Specifically, we evaluated whether pre-ECT data and post-ECT data from the same session projected into different regions of low-dimensional space. Differences between pre-ECT and post-ECT data were interpreted as reflecting ECT-related EEG changes across the available electrodes; in other words, latent patterns in the data indicating meaningful morphological (electrophysiological waveform) influences, and thus a measurable neurophysiological impact of ECT.

**Figure 2:**
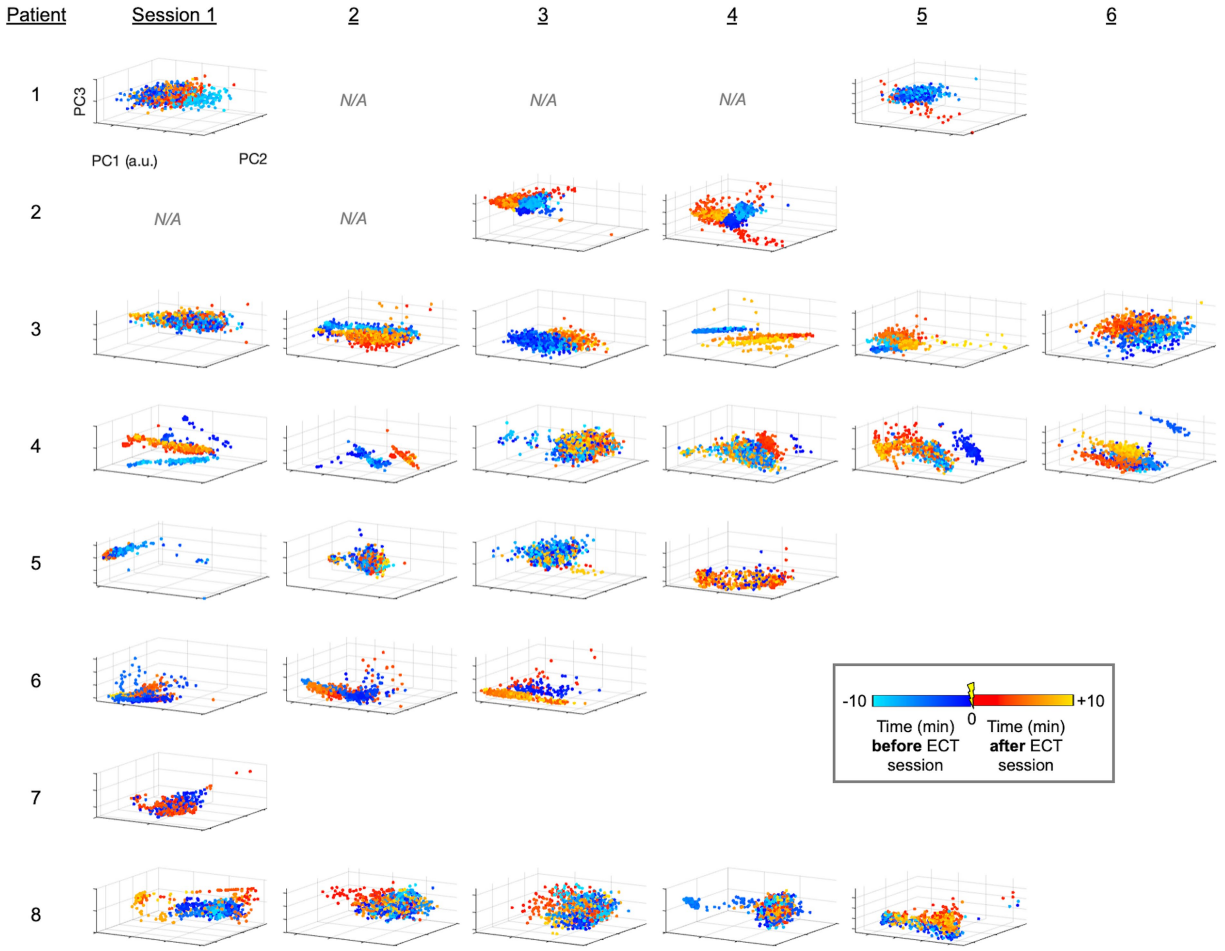
PCA-derived embeddings plots of linelength-converted EEG data for all patients and sessions. Using the workflow illustrated in Figures 1C,E, data points for each one-second interval is projected into 3-D space with axes corresponding to the first 3 PC’s. Colors designate the timing of the data points relative to ECT (legend). Patients had variable numbers of sessions (Table 1; Figure 1B). Sessions without available EEG data (i.e., deleted, see Methods) are denoted as N/A, and a.u. indicates arbitrary units (generated separately by each PCA analysis). Patients 1–4 and 5–8 are those who survived or did not survive as outcomes, respectively.

**Figure 3:**
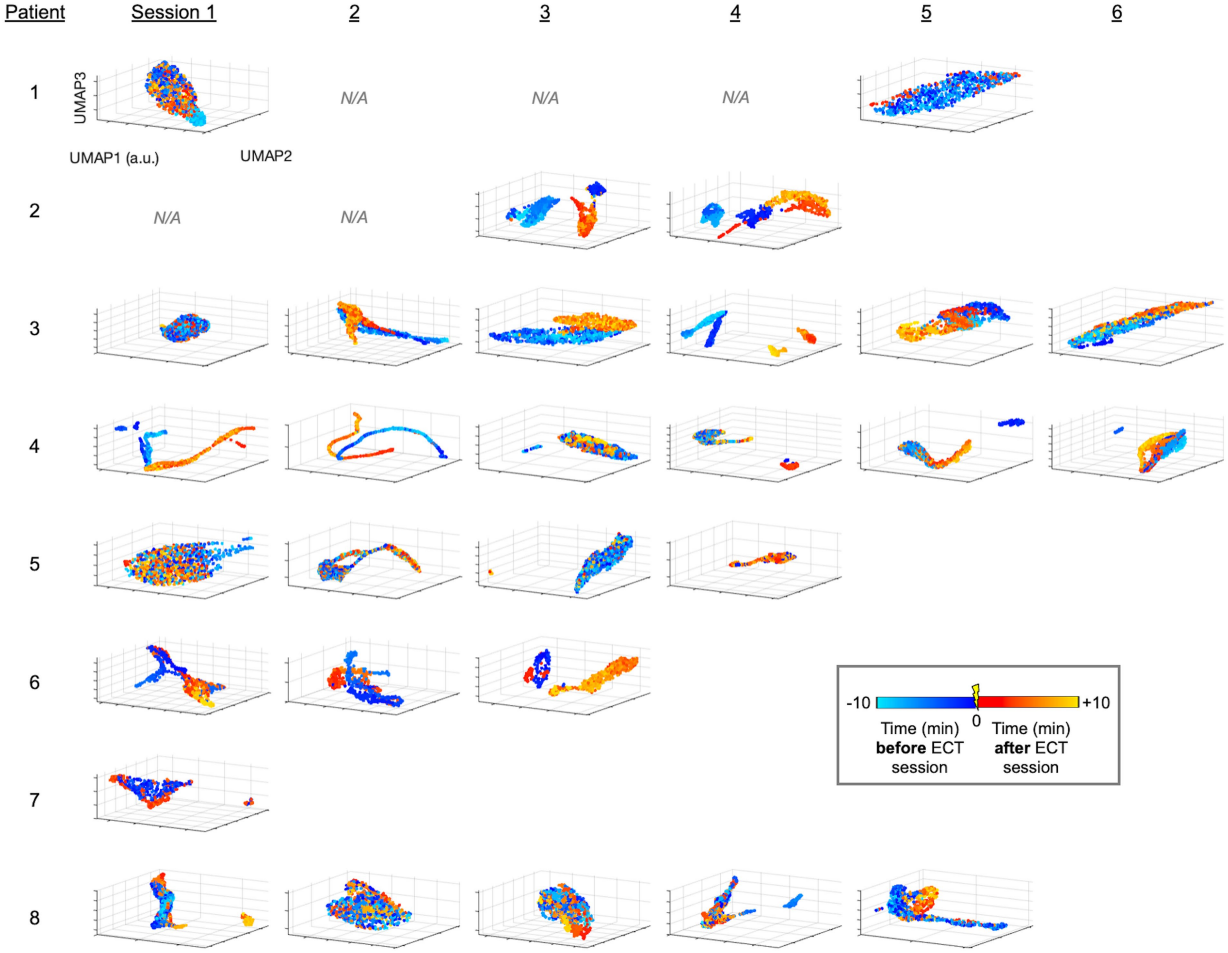
UMAP-derived embedding plots of linelength-converted EEG data for all patients and sessions. Data is projected similar to Figure 2, instead using the non-linear approach of UMAP.

**Figure 4:**
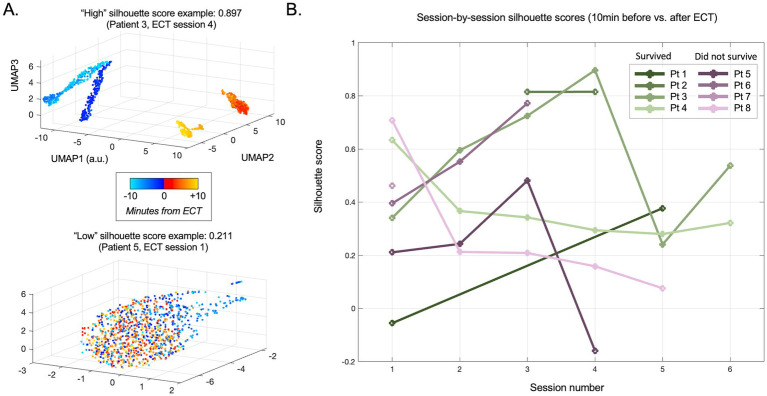
Evolution of pre- vs. post-ECT silhouette scores across sessions (days). **(A)** Individual session examples of high (upper panel) and low (lower panel) silhouette scores for data comparing 10 min immediately before vs. after ECT. Note the clear separation between nearly all pre-stimulation data points (turquoise to blue) and post-stimulation data points (red to orange) in the upper panel, suggesting an immediate and persistent difference in the scalp EEG linelength measure (surrogate for epileptiform activity) after ECT is delivered. Data in the lower panel is largely overlapping, suggesting poor separability and thus minimal no change related to ECT during this session. **(B)** Silhouette scores for all evaluated sessions across patients.

**Figure 5:**
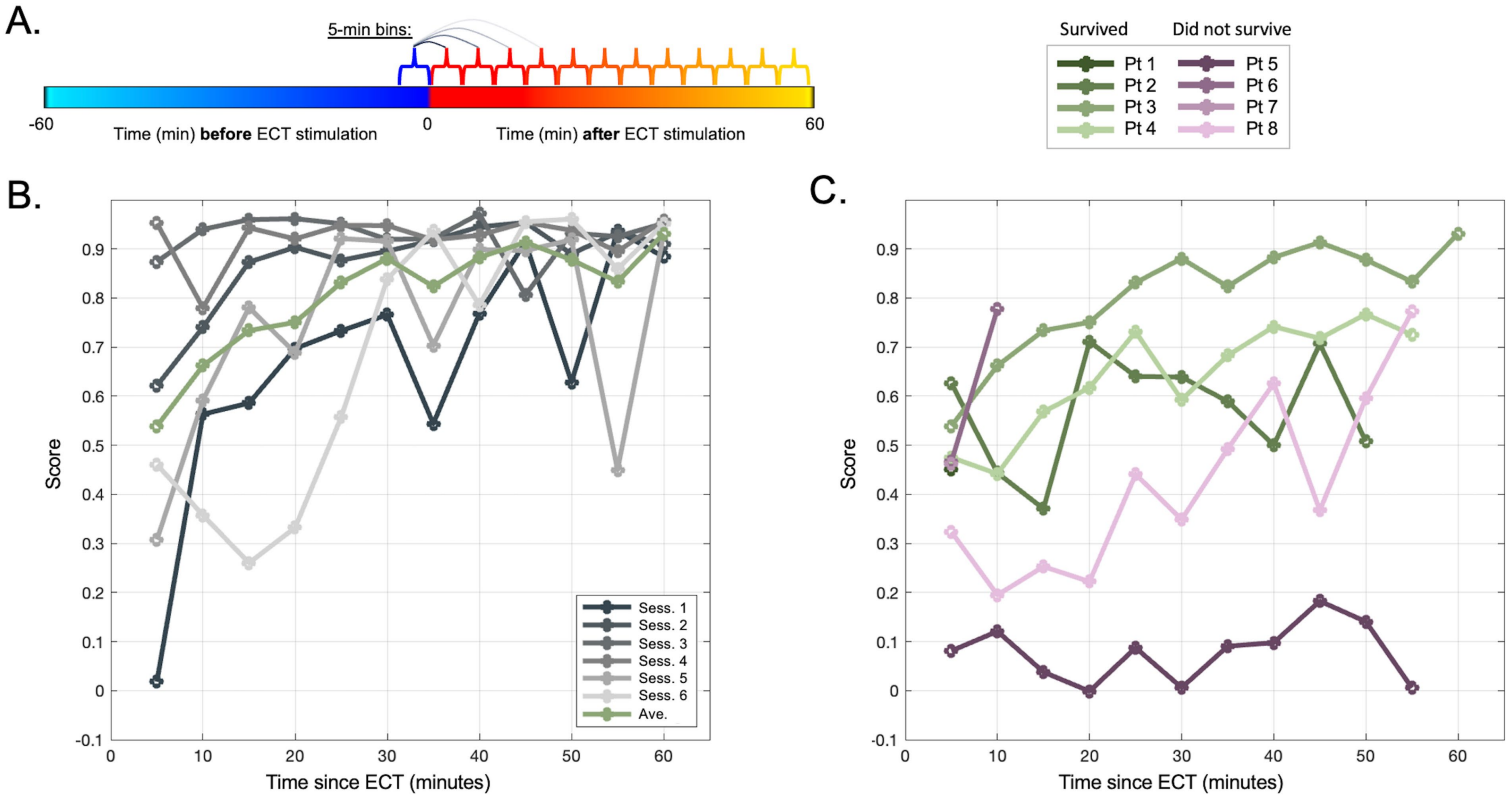
Evolution of pre- vs. post-ECT silhouette scores up to 60 min immediately post-ECT. **(A)** Schematic of analysis in panel **(B,C)** in which, for each session, consecutive 5-min periods of data are each compared to the same baseline of 5 min immediately preceding ECT. **(B)** Silhouette scores for each session (gray traces) of an example patient (Pt 3) illustrating the estimated separability between pre-ECT segment [blue period in panel **(A)**] vs. post-ECT segments over time [consecutive red-orange segments in panel **(A)**]. Green traces illustrate the average of all sessions for this patient. **(C)** *Silhouette* score means (across sessions) similar to green trace in panel **(A)**, shown for all patients (note Pt 1 and Pt 7 restricted to 5-min mark due to limited post-ECT data across sessions, Figure 1B).

Following unsupervised analysis using PCA and UMAP on the unlabeled data, labels were revealed (colors in Figures 2, 3). We calculated silhouette scores (30) (*silhouette.m* function in MATLAB) for each ECT session using these labels in order to quantify changes in the EEG data associated with ECT. Silhouette scores ranged from −1 to +1, with a high score conceptually suggesting good separability between the portions of data (i.e., between pre-ECT and post-ECT) in low-dimensional space. Silhouette scores were calculated from the first three dimensions of the blinded PCA (or UMAP) embedding using the revealed labels (i.e., “unblinding”). These scores were compared in two temporal scenarios: (1) across consecutive sessions, and (2) as time elapsed after ECT stimuli, or in other words, across consecutive five-minute periods of post-ECT data. Specifically, in the first scenario we calculated silhouette scores for data up to 10 min before and 10 min after each ECT session for all consecutive sessions (Figure 4). In the second scenario we independently compared the 5-min period pre-ECT repeatedly to consecutive 5-min periods post-ECT, for up to 1 h (Figure 5).

We examined the association between ECT and silhouette scores with mixed effects models (*fitlme.m* function in MATLAB). We incorporated a patient-adjustment (“random effect”) in the models to account for patient-specific differences, and fixed effects of time (across sessions for the first analysis, and over time elapsed since ECT for the second analysis) and in-hospital survival (referred to hereafter as survived vs. did not survive). Since the distributions of the primary outcome data (silhouette scores of unsupervised data reductions comparing before vs. after ECT) and their global structure were unknown and variable, the above analyses were performed using PCA-transformed data as a linear dimensional reduction approach, and separately using the UMAP data as a complementary nonlinear approach.

## Results

We identified eight patients who met the eligibility criteria for inclusion. They ranged from 19 to 67 years old, and four (50%) were female (Table 1). Patients were treated with up to four anesthetic agents during their SRSE hospitalization, and up to seven anti-seizure medications not including anesthetics (Supplementary Figure 1; Table 1). Half of the patients survived, whereas the other half did not. The surviving patients had chronic localization-related epilepsy and cognitive impairments at 90 days and one-year follow-up timepoints after onset of SRSE (Table 1). Patients underwent 3–12 sessions of ECT during their hospitalization, and electrical stimuli were delivered two to four times per session. Analysis was limited to data from the first six sessions for each patient (Figure 1B; Table 1) since only two patients had additional sessions. Of the 29 ECT sessions included in the analysis, 4 (13.8%) had a change in anesthesia during the period analyzed (10 min before and 10 min after ECT) and 25 (86.2%) did not have a change in the dose of anesthesia.

### Unsupervised analysis

Briefly, we next quantified patterns of EEG changes due to ECT. To extract EEG fluctuations associated with epileptiform activity, we first applied a linelength transform to the EEG data. The high-dimensional linelength-transformed EEG channel data from individual sessions was next projected into a low-dimensional space using PCA. We observed in several ECT sessions that EEG fluctuations before treatment occupied a different region of the three-dimensional PC space than fluctuations after treatment (Figure 2). This suggests that ECT may effect scalp EEG signal composition more in some patients (patients 2 and 3) compared to others (e.g., patients 2 and 3). We next applied UMAP (20, 21, 31) to this same data, again demonstrating several sessions with overt visualizable differences before vs. after ECT, similar to PCA yet with more salient clustering (Figure 3).

### ECT-related electrographic changes: across sessions

The silhouette score provided an approximation of the degree to which the EEG signal composition changed before vs. after ECT in a given session. The use of a silhouette score thus helped to control for pre-existing differences between patients and sessions (e.g., different combinations of anesthetics and ASMs administered, different etiologies). Patients were receiving anesthesia during this period (Supplementary Figure 1) which could affect scalp EEG signal composition, however, only four sessions involved a change in anesthesia during the period analyzed. Mean silhouette scores for these sessions were in similar ranges as sessions with no dynamic anesthesia change, suggesting these differences did not confound our results (Supplementary Figure 2).

The mean silhouette score across all ECT sessions was 0.364 (median 0.294, range −0.131 to 0.943). On visual inspection, the distinction of EEG data before vs. after ECT varied widely both within and between patients, with some sessions with overlapping and others showing separation. This is reflected in variability among silhouette scores in Figure 4. A mixed effect model using the PCA-derived data (Figure 2) showed no effect of consecutive session (OR: −0.009, CI: −0.078 to 0.060, *p* = 0.795), nor patient survival (OR: −0.101, CI: −0.117 to 0.319, *p* = 0.349) on silhouette scores. Replicating the mixed effect model using UMAP-derived data as a non-linear data reduction approach (Figures 3, 4) also showed no effect of consecutive session (OR: −0.025, CI: −0.082 to 0.032, *p* = 0.374) or outcome (OR: 0.149, CI: −0.111 to 0.410, *p* = 0.249).

### ECT-related electrographic changes: within sessions

The plot of silhouette scores over time within each ECT session demonstrated that the distinction between EEG data before vs. after ECT also appeared to change minute-by-minute (migrating locations of color shades in Figures 2, 3 denote EEG changes over consecutive time) suggesting evolution in the hyperacute and acute neurophysiological influences of ECT. A mixed effects model assessing consecutive 5-min bins of data (Figure 5) suggested that silhouette scores significantly increased across this 60-min post-ECT timeframe (OR: 0.003, CI: 0.001–0.005, *p* = 0.008), and scores were higher for patients who survived (OR: 0.162, CI: 0.006–0.318, *p* = 0.042). Replicating the analysis with UMAP-derived data similarly showed a positive effect across consecutive 5-min bins (OR: 0.005, CI: 0.003–0.006, *p* < 0.001), though the effect of patient outcome became a trend that did not reach statistical significance (OR: 0.217, CI: −0.044 to 0.479, *p* = 0.102).

## Discussion

ECT has shown promise in limited retrospective case reports and series that have studied clinical outcomes. Yet to our knowledge, prior reports of whether ECT treatment is associated with measurable electrographic changes are exceedingly rare. This is presumably due to challenges of reliably measuring the complex and evolving SRSE epileptiform burden on extended EEG recordings. These challenges can be addressed using semi-subjective clinician descriptions of the EEG background (16, 32), simplified metrics (e.g., epileptiform discharge rates), or more recently, objective methods using emerging computational tools (20, 21, 33). The current investigation follows the latter approach to quantitatively describe latent changes in electrographic activity associated with receiving ECT for SRSE.

Given the high morbidity and mortality of SRSE (2, 18), this study provides a much needed methodological step forward in investigating the effects of SRSE treatment, specifically demonstrating a potential neurophysiological impact of ECT treatment. Our findings suggest ECT may be associated with modulation of electrophysiological features, and we demonstrate this through objective assessment of the data. Specifically, our PCA and UMAP approaches were unsupervised, or in other words, blinded to whether the data came from before vs. after ECT stimuli in a given session. Our silhouette score approach subsequently unblinded this transformed data, evaluating the different locations in these low-dimensional PCA and UMAP space embeddings to measure how different the EEG epileptiform background was before vs. after ECT. Illustrating the data through colored dots supplemented our approaches with visual evidence of EEG signal changes before vs. after ECT in many sessions across patients (Figures 2, 3, 4A).

Evaluating the first 10 min post-ECT data did not reveal overt differences related to survival (Figure 4). We chose this relatively short segment initially since many sessions lacked data for longer periods immediately following ECT. However, our latter analysis showed that later timepoints post-ECT may show stronger separability from pre-ECT timepoints (higher silhouette scores). Indeed, when including data for up to 1 h after ECT (and statistically adjusting for time elapsed, missing values, and individual patients), a higher degree of evident modulation (higher silhouette score) in the one-hour period after ECT was apparent and related to a better clinical outcome (Figure 5).

The mechanism of an ECT-related effect on EEG recordings is unclear. However, we speculate that a stronger degree of potential modulation (i.e., a higher silhouette score) may imply that the brief ECT-induced seizure (Figures 1A,D) engaged larger regions of cortex and/or engaged the involved brain tissue to a greater degree. ECT-induced seizure activity superimposed on ongoing status epilepticus could affect metabolism, neurotransmitter transmission, gene expression, and/or receptor translocation (34–36). We speculate that such influences could be reflected by overall tissue neurophysiology and excitability, hence ECT-related changes in the electrographic ictal (status epilepticus-related) patterns observed in our study.

Overall, a greater before vs. after silhouette score noted in the hour after ECT may indicate susceptibility to ECT treatment and thus potentially better outcomes. However, our study was likely relatively underpowered due to the rarity of SRSE, perhaps as evidenced by the influence of outcome of silhouette scores becoming a non-significant trend when using UMAP data. A larger prospective study, ideally across multiple institutions to increase patient volumes, would be required to verify true potential efficacy of ECT for SRSE.

Our study had several potential limitations. First, the availability of data was limited, including the number of total patients given the rareness of SRSE, and availability of data since patients who were hospitalized in earlier years often lacked complete EEG recordings due to historical practices of pruning data for digital storage limits. Patients also had varying concurrent medications and doses (though we addressed these aspects with our within-subject study design; see Methods) and other differences in treatment courses (e.g., ECT stimulus duration and intensity). Such influences could affect EEG features and interact with the influence of ECT. These aspects reduce the generalizability of our findings, underscoring that future studies, particularly combining larger numbers of patients across multiple centers with specific ECT parameters, are crucially needed. Limitations in data availability were also due to artifact related to routine ICU care (24), which we omitted to prevent data skewing (25). Second, we used a single transform (linelength) as a surrogate for ictal activity given its validation as a metric for ictal and other epileptiform activity (26, 27) As the sum of the absolute voltage changes over a set time window of data points, linelength inherently incorporates common metrics of ictal severity (e.g., spike discharge rate) (37). Yet this is just one of many potential metrics, and other transforms provide various pros and cons (e.g., sensitivity and specificity for epileptiform activity) (20, 21). Our PCA and UMAP approaches were used to evaluate the data using complementary linear and non-linear clustering techniques, though other approaches such as non-negative matrix factorization and t-SNE may be relevant as well (38, 39).

## Conclusion

Our findings provide evidence for a measurable and potentially lasting effect of ECT on scalp EEG-recorded neural signals. This quantitative study adds novel insights into the understanding of whether and how ECT may influence patient variables, and thus our findings may have future implications for expanding the use of ECT in the neurocritical care setting. However, given the typically poor outcomes of SRSE, further investigation into ECT as a potential therapy is strongly warranted, ideally combining larger numbers of patients across multiple centers using a prospective randomized design (e.g., ECT vs. placebo). This study further demonstrates the potential value of quantitative EEG metrics for such evidence-based studies, so that measurable differences in treatment effects can be more objectively evaluated among emerging therapies for devastating neurological conditions.

## Data Availability

The datasets presented in this article are not readily available. The de-identified data and MATLAB-based code to reproduce the results in this study is available upon reasonable request and upon approval of a data access agreement to protect against re-identification in light of the limited cohort size and sensitive nature of patient data. Requests to access the datasets should be directed to jon.kleen@ucsf.edu.
