# Supplementary figures and images for: Quantitative evaluation of electrographic response to electroconvulsive therapy in super-refractory status epilepticus
Source: Front Neurol. 2024 Dec 16;15:1493336. doi: 10.3389/fneur.2024.1493336 (PMC11688648; doi:10.3389/fneur.2024.1493336)

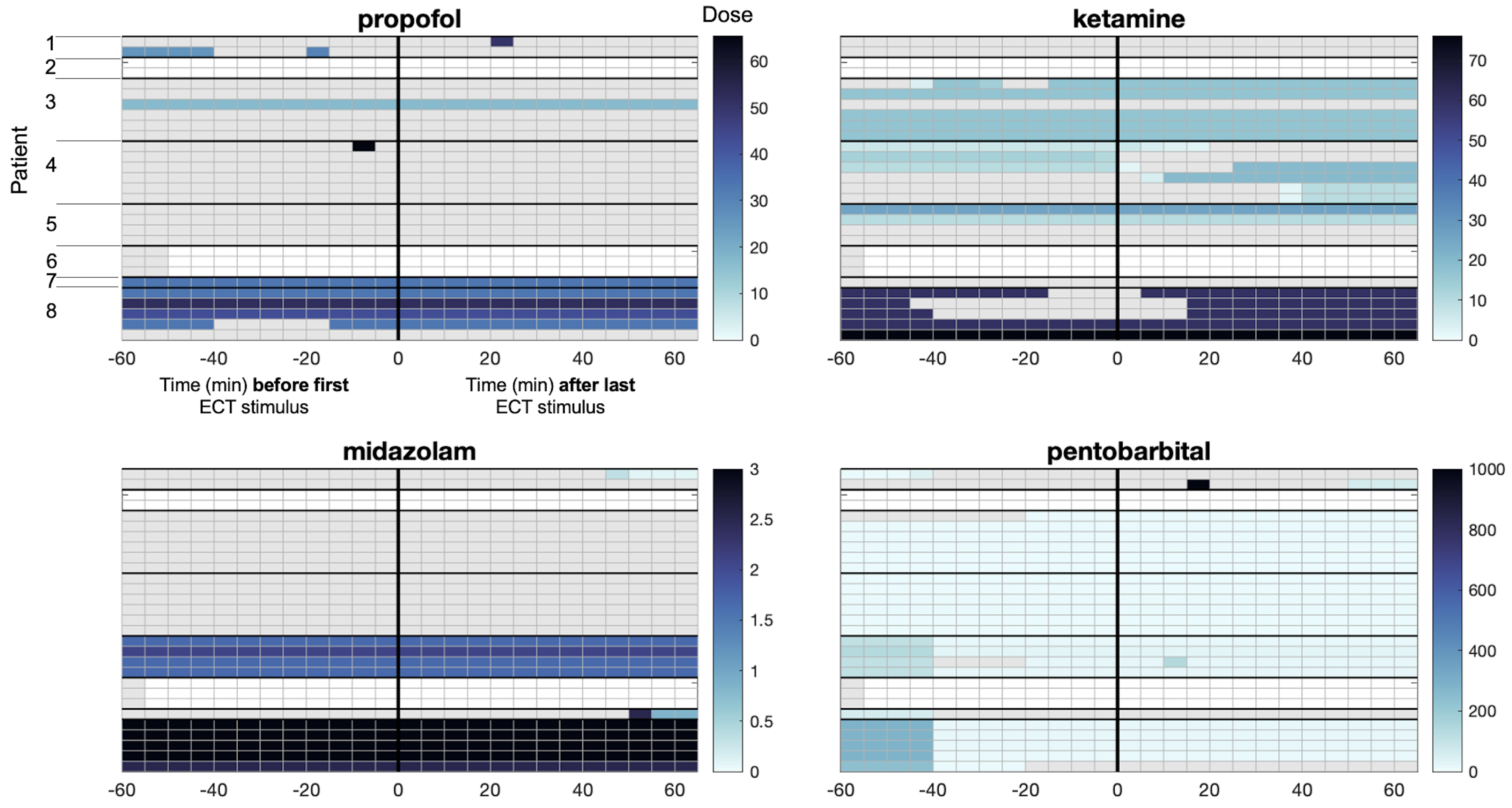

Supplement: Supplementary file 1 [file Image_1.TIFF]

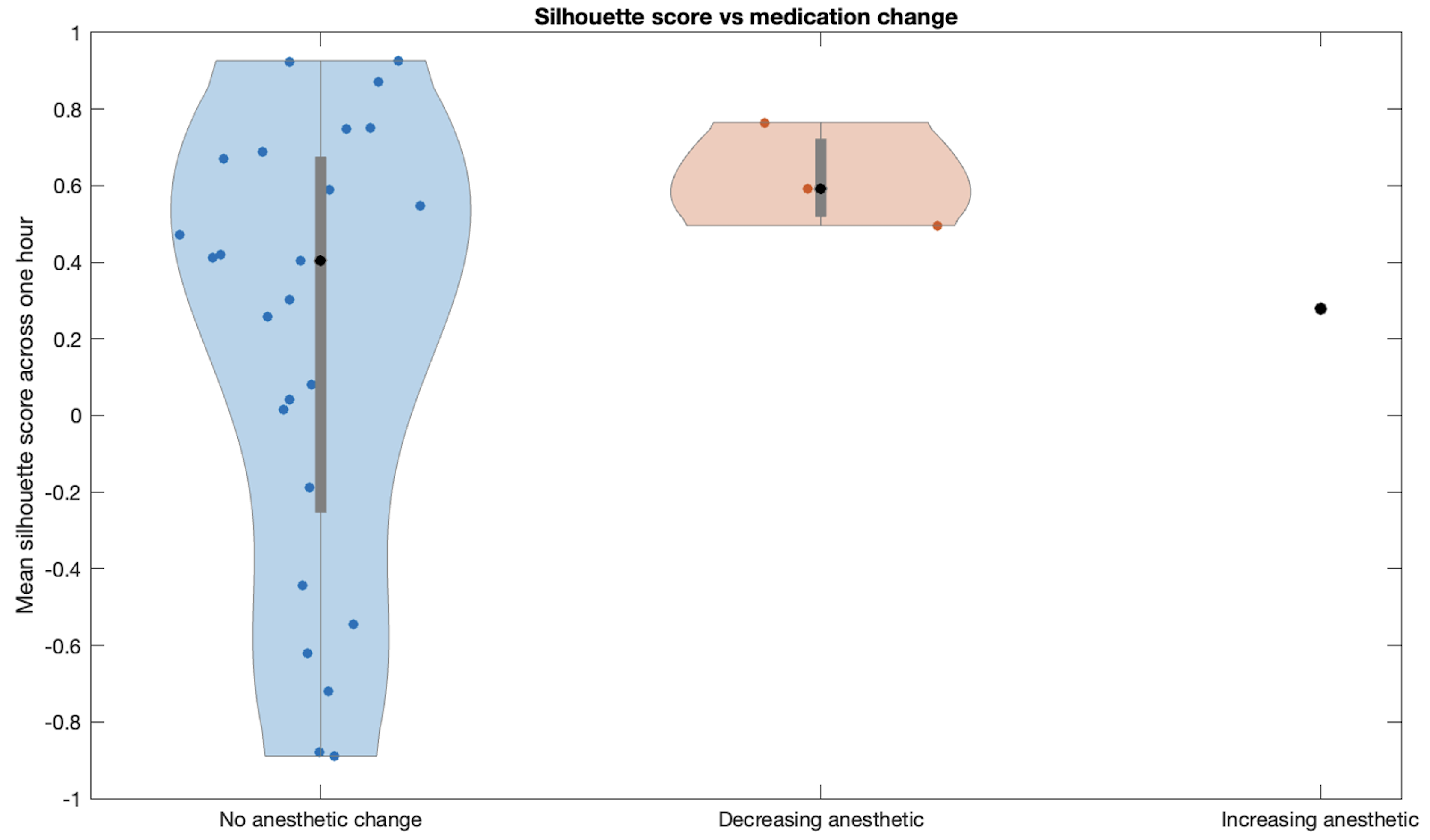

Supplement: Supplementary file 2 [file Image_2.TIFF]
