# Supplementary material for: Quantitative evaluation of electrographic response to electroconvulsive therapy in super-refractory status epilepticus
Source: Front Neurol. 2024 Dec 16;15:1493336. doi: 10.3389/fneur.2024.1493336 (PMC11688648; doi:10.3389/fneur.2024.1493336)
Supplement: Supplementary file 3 [file Data_Sheet_1.PDF]

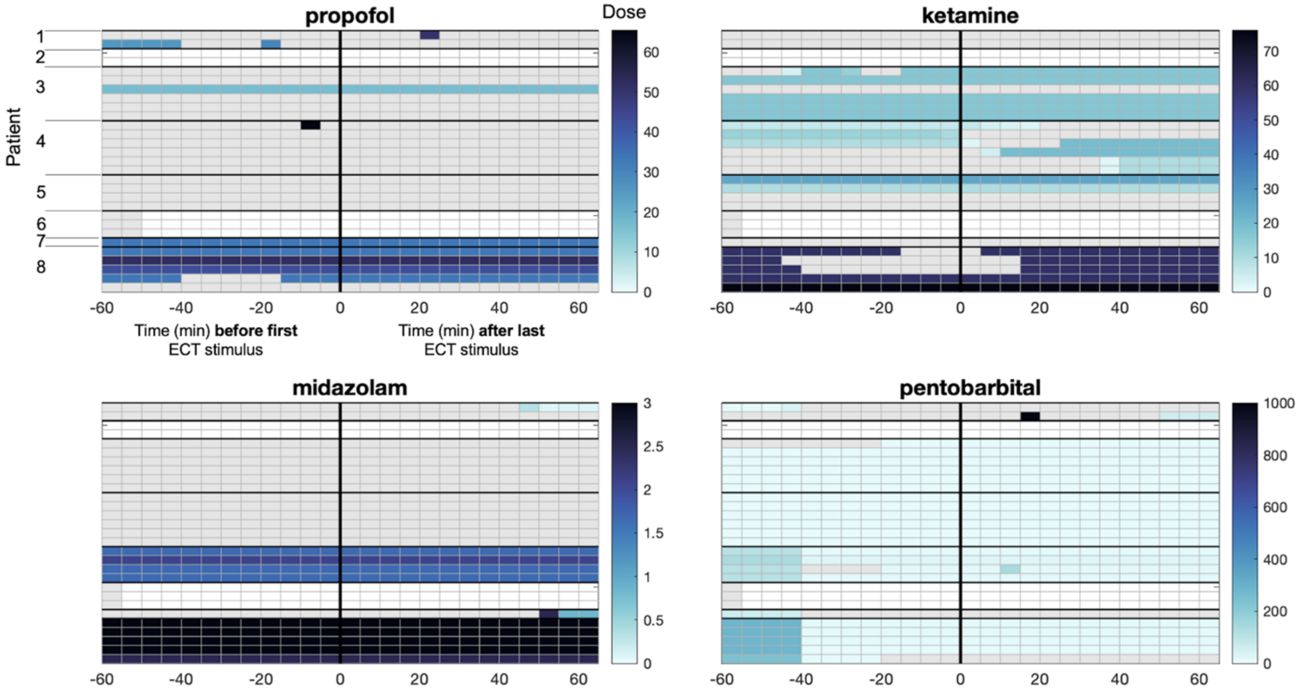

Supplementary Figure 1. Drug dosages over time for ECT sessions analyzed herein (milligrams per 5-min interval).

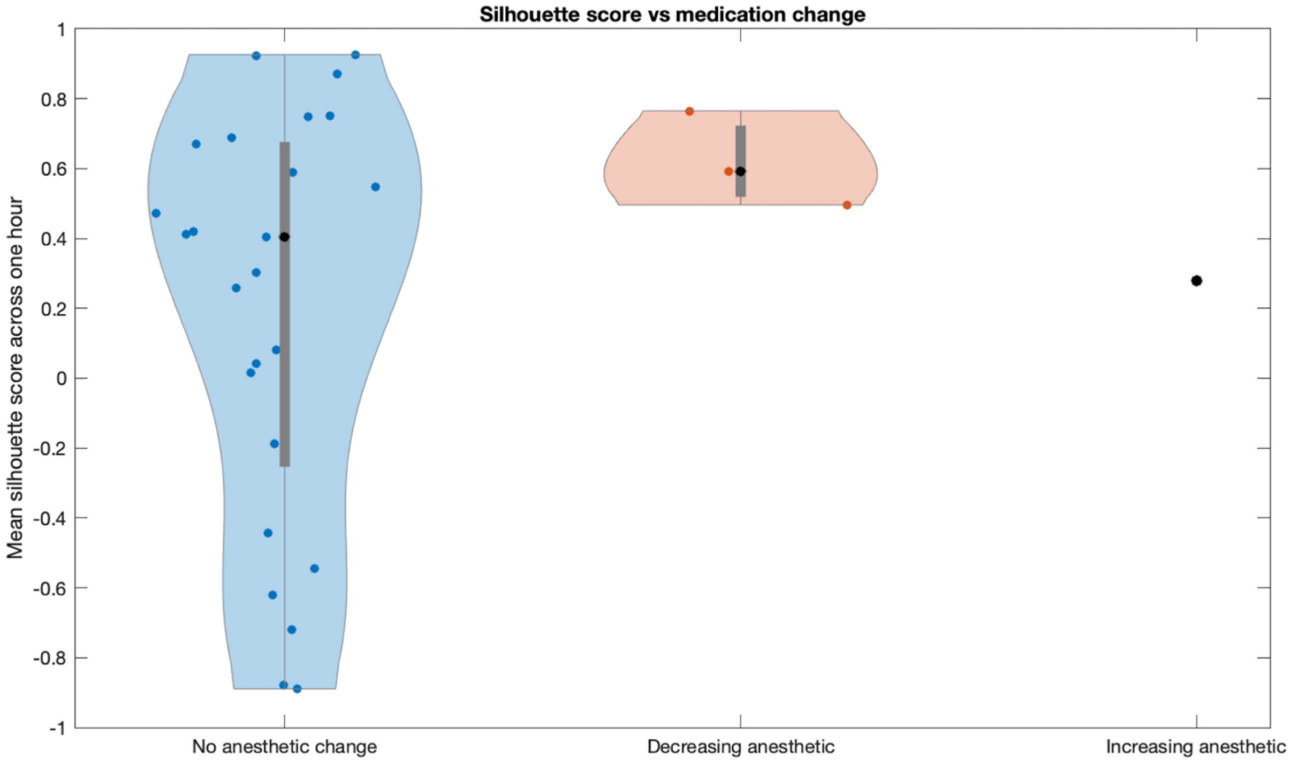

Supplementary Figure 2. Drug dosages over time for ECT sessions analyzed herein (milligrams per 5-min interval).
